# Evaluation of Triamcinolone Acetonide and Curcumin Lozenges in Patients With Erosive Lichen Planus: A Pilot Clinical Study

**DOI:** 10.7759/cureus.90574

**Published:** 2025-08-20

**Authors:** Navin Mishra, Md Jawed Iqbal, Sheeri Sabir, Sufia Parveen

**Affiliations:** 1 Department of Conservative Dentistry and Endodontics, Post Graduate Institute of Dental Education and Research (PGIDER) Indira Gandhi Institute of Medial Sciences, Patna, IND; 2 Department of Dentistry, Patna Medical College and Hospital, Patna, IND; 3 Department of Public Health Dentistry, Career Post Graduate Institute of Dental Sciences and Hospital, Lucknow, IND; 4 Department of Conservative Dentistry and Endodontics, Buddha Institute of Dental Sciences and Hospital, Patna, IND

**Keywords:** anti-inflammatory, corticosteroids, curcumin, herbal, oral erosive lichen planus

## Abstract

Background

Oral erosive lichen planus is an autoimmune-mediated mucocutaneous disorder that manifests as painful ulcerative lesions present bilaterally on the buccal mucosa, tongue, and lips. To date, there is no single definitive treatment for oral lichen planus (OLP). Corticosteroids have been the mainstay of treatment; however, their usage is associated with potential side effects. Curcumin, a plant-based herbal medicament, can be a safer alternative to these synthetic drugs. Hence, the aim of the present study was to compare and evaluate the effect of curcumin lozenges and topical corticosteroids for the treatment of erosive lichen planus lesions.

Material and methods

Twenty patients were included in the study and were randomly divided into two groups. Patients in Group 1 were given triamcinolone acetonide 0.1% for topical application along with multivitamin capsules, and patients in Group 2 were given turmeric lozenges (Turmnova, Gelnova Laboratories (India) Pvt. Ltd., Navi Mumbai, India) along with multivitamin capsules. Improvement in pain intensity was recorded on the Visual Analogue Scale (VAS) at baseline and on subsequent visits after one week and two weeks, and clinical healing was evaluated after two weeks. All the data collected were analyzed using IBM SPSS Statistics software, version 22 (IBM Corp., Armonk, NY) using the Mann-Whitney test, where p<0.05 was considered statistically significant.

Results

In our study, curcumin lozenges have shown similar improvement in the severity of pain after one week and two weeks when compared with triamcinolone acetonide 0.1% paste. Curcumin has shown promising results by decreasing the lesion size, pain, and burning sensation. Hence, it can be a new ray of hope in minimizing the signs and symptoms of OLP with minimal side effects.

## Introduction

Oral lichen planus (OLP) is a relatively common chronic, immune-mediated inflammatory disorder that affects both the skin and mucous membranes. It presents in various clinical forms, ranging from keratotic (reticular or plaque-like) to erythematous, ulcerative, and erosive lesions [1]. First described by Wilson in 1869, OLP affects approximately 0.5% to 2% of the global population. In most cases, oral lesions are accompanied by cutaneous manifestations. The condition is more prevalent among middle-aged women and is rarely seen in children or adolescents [2]. Cutaneous lesions typically appear bilaterally and symmetrically, commonly involving the flexor surfaces of the wrists and forearms, as well as the trunk, inner thighs, and knees. In the oral cavity, OLP often presents as bilateral lesions, most frequently on the buccal mucosa, tongue, lips, and gingiva, and less commonly on the floor of the mouth and palate. These oral lesions are typically described as grayish-white, velvety, thread-like papules arranged in linear, annular, or reticular patterns, often forming a distinctive lacy appearance on the buccal mucosa [3]. The erosive form of OLP is considered to result from autoimmune-mediated damage, predominantly involving cytotoxic CD8+ T cells that induce apoptosis in the basal epithelial cells [4]. These erosive lesions are typically painful, ulcerative, and symmetrically distributed on the buccal mucosa, lips, and tongue. The disease course is often chronic, marked by phases of remission and exacerbation, which may be triggered by stress, trauma, infections, emotional disturbances, or nutritional deficiencies. Patients frequently experience mucosal bleeding with minor trauma, such as tooth brushing or eating, which can lead to anorexia, weight loss, nutritional compromise, and even psychological distress, including depression [3]. Currently, there is no universally accepted definitive treatment for erosive OLP. The primary therapeutic goals are to relieve symptoms, promote healing of lesions, extend symptom-free intervals, and maintain good oral hygiene. Treatment options include corticosteroids, calcineurin inhibitors, retinoids, dapsone, hydroxychloroquine, mycophenolate mofetil, enoxaparin, photodynamic therapy, and carbon dioxide (CO₂) laser therapy [5]. Among these, corticosteroids remain the first-line therapy due to their immunosuppressive efficacy in controlling T-cell-mediated inflammation. However, long-term use of corticosteroids, whether topical or systemic, can lead to several adverse effects, such as mucosal thinning, systemic absorption, gastrointestinal irritation, adrenal suppression, opportunistic fungal infections, and general toxicity [6]. Given these limitations, the use of natural herbal remedies has garnered attention due to their lower side-effect profiles. One such compound is curcumin, a bioactive ingredient extracted from the *Curcuma longa* plant, known for its potent antioxidant, anti-inflammatory, antimicrobial, and anticancer properties [7]. Hence, the aim of the present study was to compare and evaluate the effect of curcumin lozenges and topical corticosteroids for the treatment of erosive OLP lesions.

## Materials and methods

This study was conducted in the Department of Conservative Dentistry and Endodontics, Postgraduate Institute of Dental Education and Research, Indira Gandhi Institute of Medical Sciences (IGIMS), Patna, Bihar, India. It adhered strictly to the ethical principles outlined in the Declaration of Helsinki and was approved by the Institutional Ethics Committee (IEC) following an independent peer review (IEC No: 1747/IEC/IGIMS) prior to the initiation of the research.

Before enrollment, all participants, or their parents in cases involving individuals under 14 years of age, were provided with detailed information about the treatment protocol as outlined in the patient information sheet. Written informed consent was obtained from each participant accordingly.

This prospective clinical study enrolled twenty patients presenting with clinical features of erosive OLP, who were selected from the outpatient department. Participants were randomly assigned into two groups: Group 1 received topical triamcinolone acetonide 0.1% along with a daily becadexamin capsule, while Group 2 was administered curcumin lozenges in combination with the same nutritional supplement (becadexamin capsule). This study was conducted from June 2023 to March 2025.

Sample size calculation

The required sample size for this study was calculated using the G*Power software (version 3.1.9.7; Heinrich-Heine-Universität, Düsseldorf, Germany), a free and widely used tool for statistical power analysis. The calculation was based on an equivalence margin of 0.5, a standard deviation of 0.73, a significance level (α) of 0.05, 85% statistical power, and a two-tailed hypothesis. To further ensure adequate power and account for possible dropouts, the calculated sample size was increased by 18%. Accordingly, a total of 20 participants were enrolled in this pilot clinical study.

Inclusion criteria

The study included patients presenting with clinical signs of erosive OLP. Diagnosis was made based on characteristic clinical features consistent with the condition.

Exclusion criteria

Patients who were allergic to either corticosteroids or curcumin were excluded from the study. Additionally, pregnant and lactating women were not considered for participation. Patients with a known history of gastric or duodenal ulcers were excluded to avoid potential complications related to the interventions.

Consolidated Standards of Reporting Trials (CONSORT) flow diagram

**Figure 1:**
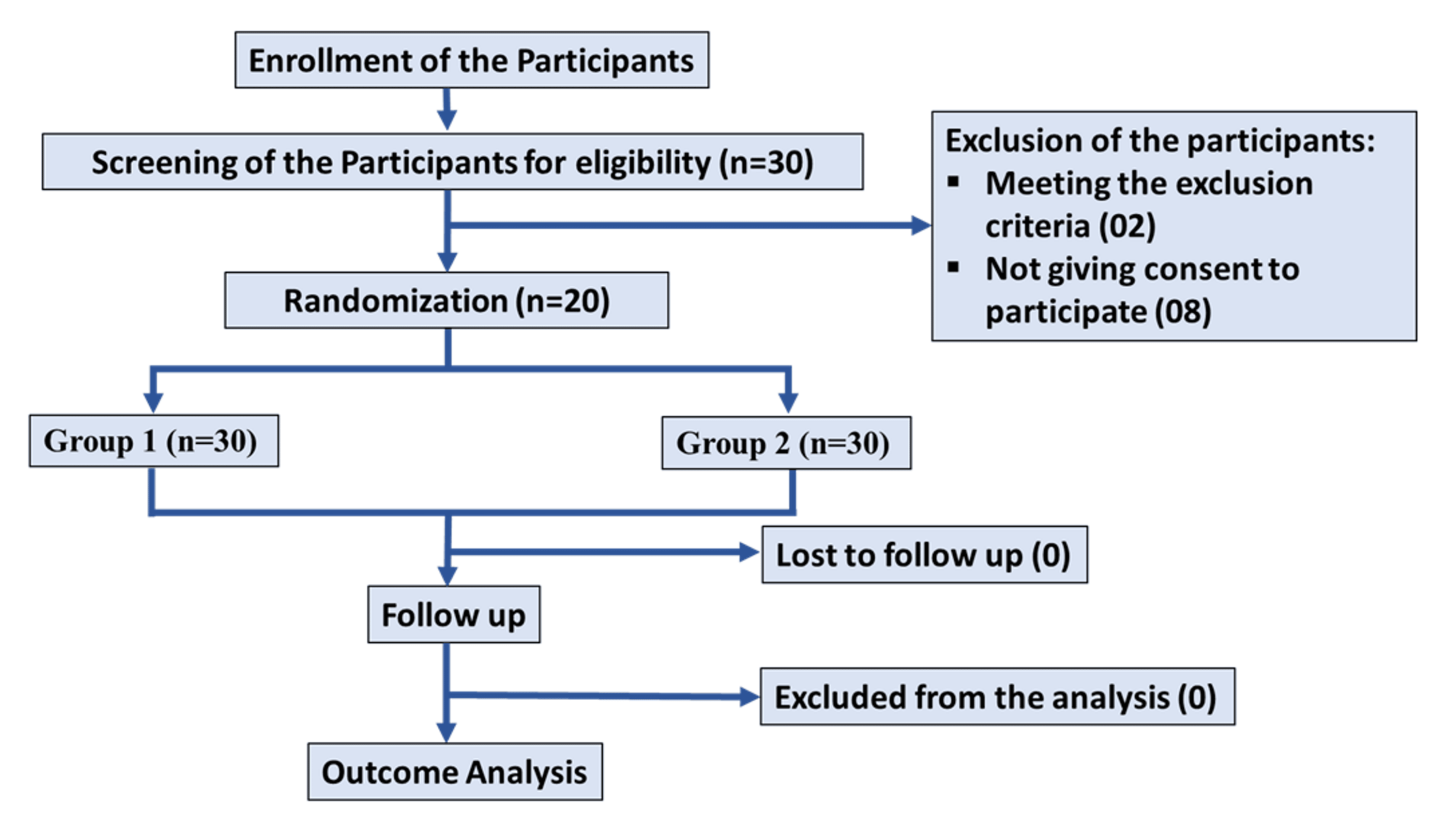
Consolidated Standards of Reporting Trials (CONSORT) flow diagram of the study participants

The CONSORT flow diagram illustrating the flow of study participants is shown in Figure 1.

The interventions used in this pilot clinical study were corticosteroid topical paste (triamcinolone acetonide 0.1% (Kenacort 0.1% paste, Abbott Healthcare Pvt. Ltd., Mumbai, India)) to apply twice daily for two weeks on the lesion in Group 1, along with a becadexamin capsule once daily for two weeks. Patients in Group 2 were given turmeric lozenges (turmeric extract 100 mg, 6.4 mg eucalyptus oil, and menthol oil 6.3 mg (Turmnova lozenges, Gelnova Laboratories (India) Pvt. Ltd., Navi Mumbai, India) three times daily for two weeks and a Beadexamin capsule once daily for two weeks. Improvement in pain intensity was recorded on the Visual Analogue Scale (VAS) at baseline and on subsequent visits after one week and two weeks. In our study, clinical healing was evaluated after two weeks. An excellent response was defined as a 100% reduction in signs and symptoms, and a very good response was defined as a 50% or more reduction in signs and symptoms, but still less than 100%. A good response was defined as less than a 50% reduction in signs and symptoms.

Clinical assessment of pain was done by VAS at baseline and was further assessed after one week and two weeks. All the data collected were analyzed using IBM SPSS Statistics software, version 22 (IBM Corp., Armonk, NY) using the Mann-Whitney test, where P<0.05 was considered statistically significant.

## Results

**Figure 2:**
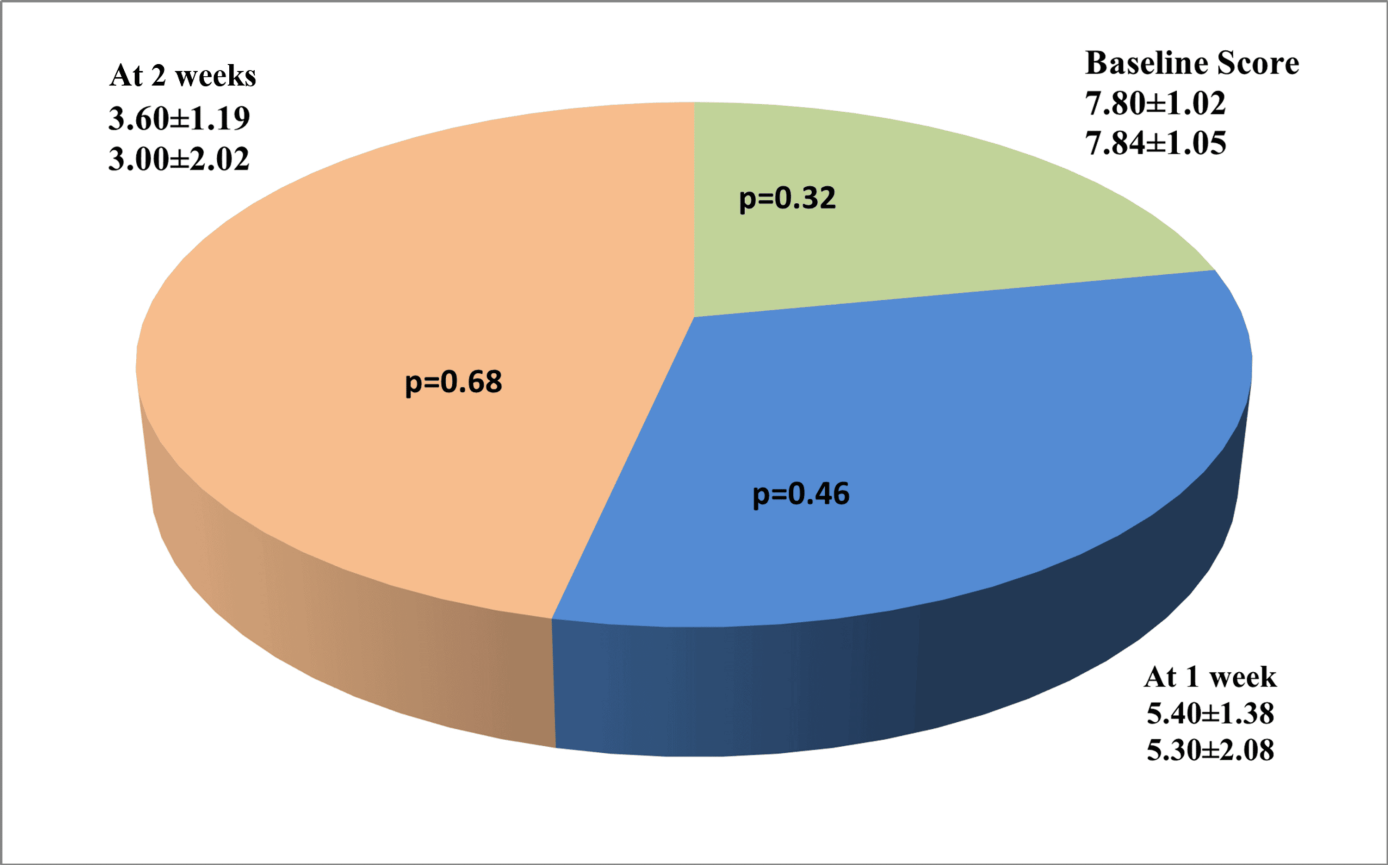
Intergroup comparison of the mean VAS scores at baseline, one week, and two weeks VAS: Visual Analogue Scale

Table 1 and Figure 2 present the intergroup comparison of mean VAS scores at baseline, one week, and two weeks. The results showed no statistically significant difference between the two treatment groups at any of the time points. In contrast, Table 2 illustrates the intragroup comparisons, which revealed a highly significant reduction in pain severity within both groups over time. Specifically, statistically significant improvements were observed from baseline to one week, baseline to two weeks, and one week to two weeks, with p-values < 0.001 for all comparisons. At the conclusion of the two-week treatment period, clinical healing was assessed in both groups. In the triamcinolone group, four out of 10 patients (40%) demonstrated an excellent response (complete resolution of symptoms), two patients (20%) had a very good response (≥50% but <100% improvement), and four patients (40%) showed a good response (<50% improvement). Similarly, in the curcumin group, five out of 10 patients (50%) exhibited an excellent response, three patients (30%) showed a very good response, and two patients (20%) had a good response. Overall, the results of this study indicate that curcumin lozenges produced comparable clinical outcomes to topical triamcinolone acetonide 0.1% paste in reducing pain severity and promoting healing in patients with erosive OLP over two weeks.

**Table 1:** Intergroup comparison of mean VAS scores at baseline, one week, and two weeks VAS: Visual Analogue Scale

Group	Baseline score	At one week	At two weeks
Group 1 (n=10)	7.80±1.02	5.40±1.38	3.60±1.19
Group 2 (n=10)	7.84±1.05	5.30±2.08	3.00±2.02
p-value*	0.32	0.46	0.68

**Table 2:** Intragroup comparison of mean VAS scores at baseline, one week, and two weeks VAS: Visual Analogue Scale

Groups	Baseline	At 1 week	At 2 weeks	p-value	p-value (Baseline vs. at 2 weeks)	p-value
				[Baseline vs at 1 week]		(At 1 week vs. at 2 weeks)
Group 1	7.80±1.02	5.40±1.38	3.60 ± 1.19	< 0.001	< 0.001	< 0.001
Group 2	7.84±1.05	5.30±2.08	3.00 ± 2.02	< 0.001	< 0.001	< 0.001

## Discussion

OLP is a chronic autoimmune disease that needs effective palliative treatment. Erosive and atrophic types of lichen planus cause burning to severe pain, which significantly affects the quality of life of the patients [8]. Malignant transformation is one of the most potent complications of OLP, but it is still controversial [9]. However, most studies indicate that OLP patients may develop oral cancer, increasing the risk of incidence (10 times) compared to the general population [10, 11]. The primary goal of treating OLP is to provide symptomatic relief for the patient and eliminate potential triggers and irritants, such as dental malocclusion, fractured carious teeth, and ill-fitting dentures.

Topical treatment is preferred due to fewer side effects, but in case of widespread lesions involving skin or other non-oral mucosa, systemic therapy is advised [12]. Most published studies consider topical corticosteroids as the most useful drug for OLP treatment, and a positive response has been seen to medium-potency corticosteroid treatment, such as triamcinolone acetate 0.1% [13, 14]. Adrenal suppression is not found in the long term, even with oral application of topical corticosteroids such as triamcinolone acetonide, fluocinolone, and clobetasol propionate [15, 16]. Corticosteroids have well-documented anti-inflammatory and anti-immune effects. Anti-inflammatory response is achieved by inhibiting the synthesis of the two main inflammatory products, prostaglandins and leukotrienes. It also suppresses the cell-mediated immunity by inhibiting genes that code for the pro-inflammatory cytokines and tumor necrosis factor-α genes [17]. Alternative treatments are retinoids, ultraviolet phototherapy, steroid-sparing agents (hydroxychloroquine, azathioprine, and mycophenolate mofetil), and pimecrolimus. These drugs have shown positive results in the treatment of OLP; however, resistance to treatment, recurrence of lesions, and a high risk of toxicities limit their use. The FDA issued a "black box" warning on the use of tacrolimus and pimecrolimus due to an increased theoretical risk of cancer (squamous cell carcinoma and lymphoma) in patients treated with tacrolimus/pimecrolimus for psoriasis [18, 19]. Pseudomembranous candidiasis and mucosal atrophy are the only common side effects of treatment with topical corticosteroids [20]. Therefore, suitable alternatives to these synthetic drugs, which may cause systemic toxicity and other side effects, are limited, especially considering the chronic nature of OLP. In our study, we have used the herbal medicament curcumin, which is nontoxic and has diversified effects in various oral diseases. It exhibits anti-inflammatory, antioxidant, antimicrobial, anti-carcinogenic, anti-proliferative, anti-mutagenic, neuro-protective, and immune-system modulating properties, which have been confirmed in many previous studies [21, 22]. The reason for the use of turmeric in our study is that in patients with OLP, there is destruction of the basement membrane by the lymphocytes, and apoptosis of inflammatory cells is also reduced or absent, which is believed to contribute to the development of OLP [23]. Thus, turmeric can induce apoptosis, promote healing, and help us reduce the severity of disease. Another mechanism of destruction is proteolytic degradation of the connective tissue matrix of the oral mucous membrane in such patients, which can also be prevented by turmeric, as it inhibits MMP-9 expression via inhibition of nuclear factor-kappa B assembly and can help in maintaining the integrity of the oral mucous membrane [24]. In most of the studies, curcumin has been used in higher doses due to rapid metabolism and rapid systemic elimination, which causes poor bioavailability due to the first-pass effect. Though curcumin is well tolerated at higher doses, it can have fewer side effects, such as abdominal discomfort, nausea, and diarrhea [25, 26]. In our study, we have used Turnova lozenges that contain the purest form of whole turmeric extract, delivered in the form of a buccal dissolving lozenge. The Quicksorb Technology patented in this product helps dissolve with our saliva, thereby keeping its bioactive ingredients intact, with all its therapeutic activity delivered into the bloodstream instantly, even at the lowest doses of 100 milligrams. Buccal absorption bypasses the hostile environment of the gastrointestinal tract. In 2012, Chainani-Wu et al. conducted a study on 20 patients where curcuminoids at doses of 6000 mg/d in 3 divided doses were given for 12 days. They concluded that curcumin was well tolerated and may prove efficacious in controlling signs and symptoms of oral lichen planus [27]. In 2015, Amirchaghmaghi et al. performed a randomized controlled trial on patients with OLP. They concluded that curcumin can be a better alternative to steroids with minimal side effects [28]. In our study, the VAS scores decreased in both groups from baseline to one-week and two-week follow-up sessions. In Table 2, the Mann-Whitney test showed that the differences between the follow-up sessions were significant (P < 0.05). However, the differences between the group treated with curcumin and the local corticosteroid were not significant (P > 0.05) (Table 1). Similar results were seen in a study conducted by Deepika et al. (2015), where OLP patients were divided, and one group was treated with triamcinolone acetonide 0.1% and the other group with commercially available topical curcumin ointment, each to be applied thrice daily for two weeks. They concluded that curcumin can be used as an alternative to steroids in the management of signs and symptoms of OLP with minimal side effects as compared to steroids with similar efficacy [29, 30].

Limitations of the study

While the study offers encouraging findings, certain aspects provide opportunities for future research. The pilot nature and small sample size allowed for focused observation but limited broad generalization; larger, multi-center studies could strengthen external validity. The short two-week follow-up provided early outcome insights, though extended monitoring would help assess long-term benefits and recurrence. Pain was measured using the widely accepted VAS, though incorporating additional objective measures could enhance reliability. Diagnosis was based on clear clinical features, and future studies may benefit from routine histopathological confirmation to further support accuracy.

## Conclusions

In our study, curcumin was found to be a safe and effective option for controlling the signs and symptoms of OLP. Herbal medicines, i.e., turmeric, can be used as an alternative to corticosteroids. Turmeric is a safe, non-toxic, effective, and economical alternative with no side effects for many traditional drugs used. Turmeric may represent a promising new approach for minimizing the signs and symptoms of OLP while maintaining a favorable safety profile. Further studies with larger sample sizes are recommended to generalize the results.

## References

[1] Sneha S, Nisa S, Mhapuskar A (2017). Curcumin - a novel ayurvedic treatment for oral lichen planus. Int J Curr Med Pharma Res.

[2] Cheng YS, Gould A, Kurago Z, Fantasia J, Muller S (2016). Diagnosis of oral lichen planus: a position paper of the American Academy of Oral and Maxillofacial Pathology. Oral Surg Oral Med Oral Pathol Oral Radiol.

[3] Rajendran R (2017). Shafer’s Textbook of Oral Pathology. 6th Edition. Shafer’s textbook of Oral Pathology. 6th ed. Rajendran.

[4] Kuo RC, Lin HP, Sun A, Wang YP (2013). Prompt healing of erosive oral lichen planus lesion after combined corticosteroid treatment with locally injected triamcinolone acetonide plus oral prednisolone. J Formos Med Assoc.

[5] Cordova P, Rubio A, Echeverria P (2014). Oral lichen planus: a look from diagnosis to treatment. J Oral Res.

[6] Bhattacharyya I, Cohen DM, Silverman Jr S (2008). Red and white lesions of the oral mucosa. Burket’s Oral Medicine Diagnosis & Treatment. 10th Edition.

[7] Ruby AJ, Kuttan G, Babu KD, Rajasekharan KN, Kuttan R (1995). Anti- tumour and antioxidant activity of natural curcuminoids. Cancer Lett.

[8] Keenan AV, Ferraiolo D (2011). Insufficient evidence for effectiveness of any treatment for oral lichen planus. Evid Based Dent.

[9] van der Meij EH, Mast H, van der Waal I (2007). The possible premalignant character of oral lichen planus and oral lichenoid lesions: a prospective five-year follow-up study of 192 patients. Oral Oncol.

[10] Lodi G, Scully C, Carrozzo M, Griffiths M, Sugerman PB, Thongprasom K (2005). Current controversies in oral lichen planus: report of an international consensus meeting. Part 2. Clinical management and malignant transformation. Oral Surg Oral Med Oral Pathol Oral Radiol Endod.

[11] Gonzalez-Moles MA, Scully C, Gil-Montoya JA (2008). Oral lichen planus: controversies surrounding malignant transformation. Oral Dis.

[12] Carrozzo M, Gandolfo S (1999). The management of oral lichen planus. Oral Dis.

[13] Al-Hashimi I, Schifter M, Lockhart PB (2007). Oral lichen planus and oral lichenoid lesions: diagnostic and therapeutic considerations. Oral Surg Oral Med Oral Pathol Oral Radiol Endod.

[14] Borba Filla J, Fontanelli A, Brown M, Naval Machado M (2016). Treatment of symptomaticoral lichen planus: a literature review. Pol Otorhino Rev.

[15] Pramick M, Whitmore SE (2009). Cushing's syndrome caused by mucosal corticosteroid therapy. Int J Dermatol.

[16] Gonzalez-Moles MA, Morales P, Rodriguez-Archilla A, Isabel IR, Gonzalez-Moles S (2002). Treatment of severe chronic oral erosive lesions with clobetasol propionate in aqueous solution. Oral Surg Oral Med Oral Pathol Oral Radiol Endod.

[17] Newton R (2000). Molecular mechanisms of glucocorticoid action: what is important?. Thorax.

[18] Lodi G, Carrozzo M, Furness S, Thongprasom K (2012). Interventions for treating oral lichen planus: a systematic review. Br J Dermatol.

[19] Becker JC, Houben R, Vetter CS, Bröcker EB (2006). The carcinogenic potential of tacrolimus ointment beyond immune suppression: a hypothesis creating case report. BMC Cancer.

[20] Thongprasom K, Luangjarmekorn L, Sererat T, Taweesap W (1992). Relative efficacy of fluocinolone acetonide compared with triamcinolone acetonide in treatment of oral lichen planus. J Oral Pathol Med.

[21] Chainani-Wu N (2003). Safety and anti-inflammatory activity of curcumin: a component of tumeric (Curcuma longa). J Altern Complement Med.

[22] Jurenka JS (2009). Anti-inflammatory properties of curcumin, a major constituent of Curcuma longa: a review of preclinical and clinical research. Altern Med Rev.

[23] Roopashree MR, Gondhalekar RV, Shashikanth MC, George J, Thippeswamy SH, Shukla A (2010). Pathogenesis of oral lichen planus-a review. J Oral Pathol Med.

[24] Clark CA, McEachern MD, Shah SH (2010). Curcumin inhibits carcinogen and nicotine-induced mammalian target of rapamycin pathway activation in head and neck squamous cell carcinoma. Cancer Prev Res (Phila).

[25] Anand P, Kunnumakkara AB, Newman RA, Aggarwal BB (2007). Bioavailability of curcumin: problems and promises. Mol Pharm.

[26] Prasad S, Tyagi AK, Aggarwal BB (2014). Recent developments in delivery, bioavailability, absorption and metabolism of curcumin: the golden pigment from golden spice. Cancer Res Treat.

[27] Chainani-Wu N, Madden E, Lozada-Nur F, Silverman S Jr (2012). High-dose curcuminoids are efficacious in the reduction in symptoms and signs of oral lichen planus. J Am Acad Dermatol.

[28] Amirchaghmaghi M, Delavarian Z, Iranshahi M (2015). A randomized placebo-controlled double blind clinical trial of quercetin for treatment of oral lichen planus. J Dent Res Dent Clin Dent Prospects.

[29] Keshari D, Patil P, Guledgud MV (2015). Efficacy of topical curcumin in the management of oral lichen planus: a randomized controlled-trial. J Adv Clin Res Insight.

[30] Rasyid A, Lelo A (1999). The effect of curcumin and placebo on human gall-bladder function: an ultrasound study. Aliment Pharmacol Ther.

